# Vitamin A supplementation and risk of atopy: long-term follow-up of a randomized trial of vitamin A supplementation at six and nine months of age

**DOI:** 10.1186/1471-2431-13-190

**Published:** 2013-11-19

**Authors:** Nicholas Kiraly, Aliu Balde, Ida Marie Lisse, Helle Brander Eriksen, Peter Aaby, Christine Stabell Benn

**Affiliations:** 1 Bandim Health Project, Indepth Network, Apartado 861, Bissau, Guinea-Bissau; 2 Gastro and Food Allergy, Murdoch Childrens Research Institute, Royal Children’s Hospital, Flemington Rd, Parkville VIC 3052, Australia; 3 Research Center for Vitamins and Vaccines (CVIVA), Bandim Health Project, Statens Serum Institut, 5 Artillerivej, Copenhagen S DK-2300, Denmark

**Keywords:** Atopy, Immunization, Measles vaccine, Vitamin A supplementation

## Abstract

**Background:**

The World Health Organization recommends high-dose vitamin A supplementation (VAS) for children above six months of age in low-income countries. VAS has been associated with up-regulation of the Th2 response. We aimed to determine if VAS is associated with atopy in childhood.

**Methods:**

Infants in Guinea-Bissau were randomly allocated VAS or placebo, either at six and nine months of age, or only at nine months of age. At six months of age, children were furthermore randomized to measles vaccine or inactivated polio vaccine. At nine months of age all children received measles vaccine. Children were revisited seven years later and skin prick testing was performed. Atopy was defined as a skin prick reaction ≥3 mm.

**Results:**

40 of 263 children (15%) were atopic. Overall VAS had no significant effect on the risk of atopy (Prevalence Ratio 1.23; 95% CI 0.69-2.18). The Prevalence Ratio was 1.60 (0.66-3.90) for males and 1.00 (0.46-2.15) for females.

**Conclusions:**

There was no significant effect of VAS in infancy on atopy later in childhood. The role of infant VAS in the development of atopy is still unclear.

## Background

The World Health Organization (WHO) currently recommends high-dose vitamin A supplementation (VAS) for children over six months of age in countries with high prevalence of vitamin A deficiency. Vitamin A and its derivatives are potent immune modulators and while dietary vitamin A intake has been associated with protection from asthma in some epidemiological studies [1], there is laboratory evidence that high-dose VAS might increase atopic asthma [2,3].

In the only human study of VAS and atopy, high-dose neonatal VAS increased odds of atopy almost three-fold [4]. This effect was found at age three to nine years, although neonatal VAS had no effect on serum vitamin A levels at six weeks of age [5]. Thus, VAS given early in life may have a long-lasting imprinting effect on the developing immune system.

Here we report long-term follow-up of a randomized trial of VAS given with vaccines after six months of age [6]. The trial was originally performed to assess the adjuvant effect of VAS on measles-specific antibodies. The aim of the present follow-up study was to determine if VAS given with vaccinations after six months of age is associated with atopy in childhood.

## Methods

**Figure 1:**
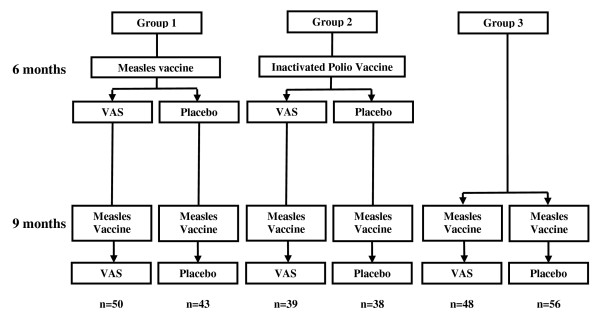
**Study profile.** VAS = Vitamin A supplementation.

The Bandim Health Project performed a randomized, double-blind, placebo-controlled trial of VAS between 1993 and 1995 in Bissau, Guinea-Bissau, as previously described [6,7]. Children approaching six months of age were visited at home in Belem and Mindara, areas covered by the Bandim Health Project’s Health and Demographic Surveillance System. Children who were <7.5 months of age were eligible for random allocation to an extra vaccine (either measles vaccine or inactivated poliomyelitis vaccine (IPV)), together with VAS or placebo (Figure 1). At nine months of age they were revisited and given a dose of measles vaccine irrespective of their original vaccination group, along with VAS or placebo as they had received at six months. Those >7.5 months of age at the first visit were not eligible for the extra vaccine but were given the usual dose of measles vaccine due at nine months of age, and were randomly allocated to VAS or placebo. VAS or placebo was given orally as 100 000 IU vitamin A or placebo in 1 mL vegetable oil with 40 IU vitamin E.

All 462 children enrolled in the original trial were eligible for follow-up. Children were revisited at home between May and August 2000 and verbal consent obtained from a parent or guardian. Verbal consent was considered appropriate at the time of the study in this population with a low literacy rate and was approved by the Guinea-Bissau Ministry of Public Health. Anthropometry and environmental data was collected. Skin prick testing was performed using four aero-allergens (*dermatophagoides pterynissinus, d. farinae*, *blomia tropicalis*, and *blatella germanica*) with positive and negative controls. All tests were performed by Dr. Aliu Balde. Data were excluded from analysis if there was no response to the positive control. Atopy was defined as a positive test to any allergen ≥3 mm after subtracting the negative control.

Groups were compared using Chi squared or Kruskal-Wallis tests. The effect of VAS was analyzed using Poisson regression providing prevalence ratios (PR) with adjustment for sex and age at follow-up. Statistical analyses were performed using Stata 11. The study received ethical approval from the Ministry of Public Health, Guinea-Bissau.

## Results

Of the 462 children enrolled in the original trial, 274 (59%) had skin prick testing performed at follow-up. 170 children received an extra vaccine and therefore also received two doses of VAS or placebo (Figure 1). 137 children received VAS and 137 children received placebo. There were no significant differences between the VAS and placebo groups at enrolment in background demography or anthropometry (Table 1).

**Table 1:** Demographics, anthropometry and prevalence of atopy according to randomization group

	**VAS (n = 137)**	**Placebo (n = 137)**	**P value or PR (95% CI)**
At enrolment			
Age (days)	200 (183–294) n = 137	198 (183–301) n = 137	0.93
Weight (kg)	7.1 (6.1-8.7) n = 88	7.5 (5.9-8.8) n = 79	0.35
MUAC (mm)	144 (126–160) n = 86	144 (130–166) n = 80	0.99
Breastfeeding	86/94 (91%)	80/90 (89%)	0.55
DTP3 vaccinated	84/137 (61%)	95/137 (69%)	0.16
At follow up			
Age (years)	7.4 (7.0-7.7) n = 137	7.4 (7.0-7.7) n = 137	0.72
Weight (kg)	20.0 (17.0-24.0) n = 136	20.5 (17.0-23.5) n = 137	0.78
Height (cm)	119 (112–126) n = 137	118 (111–125) n = 137	0.46
MUAC (mm)	166 (150–184) n = 137	166 (152–180) n = 137	0.77
BCG scar	105/132 (80%)	110/131 (84%)	0.35
Pig at the house	43/134 (32%)	47/133 (35%)	0.57
Atopy in all children (n = 263)	22/131 (17%)	18/132 (14%)	1.23 (0.69-2.18)
Atopy stratified by sex			
Boys (n = 135)	11/67 (16%)	7/68 (10%)	1.60 (0.66-3.90)
Girls (n = 128)	11/64 (17%)	11/64 (17%)	1.00 (0.46-2.15)

VAS = vitamin A supplementation; PR = prevalence ratio adjusted for age and sex; MUAC = mid-upper arm circumference; DTP3 = 3rd dose of diphtheria-tetanus-whole cell pertussis vaccine due at 14 weeks of age.

10 children had missing skin prick test data and one child did not respond to the positive control. Of the 263 children for whom valid skin prick tests were available, 40 (15%) were atopic. 19 had reactions to *d. pterynissinus*, 12 to *d. farinae*, 21 to *b. tropicalis*, and three to *b. germanica*.

Overall, VAS had no significant effect on atopy (PR 1.23; 95% CI 0.69-2.18, Table 1). The PR was 1.60 (0.66-3.90) for males and 1.00 (0.46-2.15) for females (p = 0.43 for the same effect VAS in males and females). There were no differences in the effect of VAS on atopy between groups receiving measles vaccine, IPV or no vaccine at 6 months of age (data not shown). Compared to placebo, the PR of atopy associated with one dose of VAS was 1.18 (0.45-3.15) and with two doses of VAS was 1.22 (0.60-2.49; p = 0.96 for the same effect of one and two doses of VAS).

## Discussion and conclusions

This study is the first to evaluate the effect on atopy of the WHO recommended policy of providing VAS together with vaccinations after six months of age. VAS had no significant effect on atopy later in childhood.

We recently reported a 2.8-fold increase in atopy associated with VAS when given at birth [4]. In the present study using a higher dose of VAS (100 000 IU versus 25 000 IU in [4]), we found no significant effect of VAS given after six months of age. A number of factors could explain these differing results. It may be that the immune system is most susceptible to an imprinting effect from exposure in the immediate post-natal period [8]. Alternatively, it may be due to co-administration with, or subsequent administration of, vaccinations. In our previous work, the harmful effect of VAS occurred largely in those who received BCG vaccination [4], whereas in the current work children received either measles vaccine or IPV. We have previously observed that VAS interacts with vaccines in relation to overall mortality [9].

Any effect of high-dose VAS in infancy on atopy much later in childhood would likely be unrelated to current vitamin A levels, which are unaffected by VAS beyond six weeks [5]. A number of animal studies have found positive associations between high-dose VAS and atopic phenotypes at the time of supplementation [2,3] but there are no laboratory studies examining the effect of high-dose VAS in infancy on atopy much later in life. Although we found no significant effect of VAS in infancy on atopy in childhood in the present study, it is plausible that VAS could have long-term effects on atopy as activation of RAR/RXR by all-trans retinoic acid has the ability to activate gene transcription and cause stable epigenetic changes in multiple immune cell linages [10].

We found a non-significant increase in atopy in the VAS versus placebo group. This study was limited by a small sample size and low prevalence of atopy, which may have caused a modest effect of VAS to be undetectable. In conclusion, the role of VAS in the development of atopy is still unclear, but with several new neonatal vitamin A trials ongoing [11], there are possibilities for testing it further.

## Abbreviations

WHO: World Health Organization; VAS: Vitamin A supplementation; IPV: Inactivated poliomyelitis vaccine.

## Competing interests

The authors declare that they have no potential competing interests.

## Authors’ contributions

CSB and PA designed and executed the original randomized trial. CSB was the primary investigator. AB was responsible for the skin prick test follow-up in collaboration with CSB and IML. NK, HBE, PA and CSB were responsible for statistical analysis and interpretation of the results. NK prepared the first draft of the paper. All authors contributed to and approved the final draft.

## Pre-publication history

The pre-publication history for this paper can be accessed here:

http://www.biomedcentral.com/1471-2431/13/190/prepub
